# Acyl-CoA synthetase-4, a new regulator of mTOR and a potential therapeutic target for enhanced estrogen receptor function in receptor-positive and -negative breast cancer

**DOI:** 10.18632/oncotarget.5822

**Published:** 2015-10-19

**Authors:** Ulises D. Orlando, Ana F. Castillo, Melina A. Dattilo, Angela R. Solano, Paula M. Maloberti, Ernesto J. Podesta

**Affiliations:** ^1^ Biomedical Research Institute, INBIOMED, Department of Biochemistry, School of Medicine, University of Buenos Aires, CABA, Buenos Aires, Argentina

**Keywords:** arachidonic acid, cancer, cell signaling, gene expression, tamoxifen

## Abstract

Although the role of acyl-CoA synthetase 4 (ACSL4) in mediating an aggressive phenotype is well accepted, there is little evidence as to the early steps through which ACSL4 increases tumor growth and progression. In this study, and by means of the stable transfection of MCF-7 cells with ACSL4 using the tetracycline Tet-Off system (MCF-7 Tet-Off/ACSL4), we identify the mTOR pathway as one of the main specific signatures of ACSL4 expression and demonstrate the partial involvement of the lipoxygenase pathway in the activation of mTOR. The specificity of ACSL4 action on mTOR signaling is also determined by doxycycline inhibition of ACSL4 expression in MCF-7 Tet-Off/ACSL4 cells, by the expression of ACSL4 in the non-aggressive T47D breast cancer cell line and by knocking down this enzyme expression in the MDA-MB-231 breast cancer cells, which constitutively express ACSL4. ACSL4 regulates components of the two complexes of the mTOR pathway (mTORC1/2), along with upstream regulators and substrates.

We show that mTOR inhibitor rapamycin and ACSL4 inhibitor rosiglitazone can act in combination to inhibit cell growth. In addition, we demonstrate a synergistic effect on cell growth inhibition by the combination of rosiglitazone and tamoxifen, an estrogen receptor α (ERα) inhibitor. Remarkably, this synergistic effect is also evident in the triple negative MDA-MB-231 cells *in vitro* and *in vivo*.

These results suggest that ACSL4 could be a target to restore tumor hormone dependence in tumors with poor prognosis for disease-free and overall survival, in which no effective specifically targeted therapy is readily available.

## INTRODUCTION

Cancer is a disease with genomic perturbation leading to dysregulation of multiple pathways within the cellular system. Oncogenic selection functions at the level of networks of gene expression and signaling pathways, while a body of data accumulated in the past years indicates that the metabolism of arachidonic acid (AA) is involved in the maintenance of survival and proliferating capacities of various types of normal and cancer cells and thus plays a role in tumor progression [1–3].

The expression of acyl-CoA synthetase 4 (ACSL4), an enzyme working in AA metabolism, has been shown to be associated with aggressiveness of several types of cancer such as colon and hepatocellular carcinoma [4–10]. We and others have demonstrated a positive correlation of ACSL4 expression and aggressiveness in breast cancer cell lines, with the highest expression found in metastatic lines derived from triple negative (estrogen-receptor-α (ERα)-negative, progesterone-receptor (PR)-negative and not overexpressing human epidermal growth factor 2 receptor (HER2) protein) tumor breast cancer (e.g. MDA-MB-231 and Hs578T) [6, 9].

Triple-negative breast cancer is a subtype that accounts for approximately 15% of breast cancer and is hence an important area of research for researchers and clinicians alike, as it exhibits poor prognosis for disease-free and overall survival and no effective therapy is readily available.

Functionally, we and others have found that ACSL4 is part of the mechanism responsible for increased breast cancer cell proliferation, invasion and migration, both *in vitro* and *in vivo* [4, 6, 9, 10]. The sole transfection of MCF-7 cells, a model of non-aggressive breast cancer cells, with ACSL4 cDNA transforms them into a highly aggressive phenotype, and their injection into nude mice has resulted in the development of growing tumors with marked nuclear polymorphism, a high mitotic index and low expression of ER and PR [4]. In addition, targeting ACSL4 in cells and in tumors has indeed proven to reverse the loss of ER expression [4]. These results are in agreement with those showing that ACSL4 expression correlates with the absence of ER in samples from human breast tumor [9] and that the expression of ACSL4 negatively controls the expression of ER during tumor growth.

Genetic analysis of different tumors over the past years has allowed the characterization of distinct molecular pathways altered during the development and progression of this disease. The idea of personalized medicine and molecular profiling for prognostic tests has led to a plethora of studies in the past 10 years, in search for genetic determinants of metastatic breast cancer. Such studies have identified gene sets, or “signatures”, whose expression in primary tumors is associated with higher risk of metastasis and poor disease outcome for the patients. Therefore, the identification of altered pathways and new therapeutic targets is critical to improve the management of a significant proportion of cancer patients.

Although the role of ACSL4 in mediating an aggressive phenotype in breast cancer is well accepted, the mechanism involved in this effect has yet to be fully elucidated. For this reason, the goal of this work was to study the signaling pathways triggered by ACSL4 overexpression which mediate cell phenotype change from mildly aggressive to highly aggressive in breast cancer cells.

Here, by means of cell models of ACSL4 overexpression or underexpression in addition to a pharmacological approach, we identify the mTOR pathway as one of the main specific signatures of ACSL4 expression. ACSL4 regulates components of the two complexes of the mammalian target of rapamycin (mTOR) pathway (mTORC1/2), along with its upstream regulators and substrates. Our findings reveal a significant increase in the phosphorylation of ribosomal protein S6 kinase 70kDa polypeptide 1 (p70S6K) on Thr389 and its substrates -the ribosomal protein S6-. An increase was also observed in the phosphorylation of Rictor (rapamycin-insensitive companion of mTOR) on Thr1135, substrate of p70S6K and component of mTORC2 complex. In addition, an enhancement was detected in AKT (protein kinase B or PKB) phosphorylation on Ser473. Glycogen synthase kinase-3 alpha and beta (GSK3α and GSK3β) phosphorylation levels on Ser21/9 also increased in response to ACSL4 expression, which inhibited GSK3 activity and therefore contributed to mTOR activation. In addition, we show here a synergistic effect in the inhibition of cell growth by a combination of ACSL4 and ER inhibitors. The combination was effective in inhibiting cell proliferation and tumor growth in a very aggressive triple negative breast cancer cell line, MDA-MB-231, which does not express ER and overexpresses ACSL4. These results suggest that ACSL4, in combination with ER inhibitors, could be an interesting target to be used in combination with other inhibitors and which might prevent the side effects of supra-maximal doses and generate more positive effects than single-drug therapy.

## RESULTS

### An ACSL4 functional proteomic signature of MCF-7 Tet-Off/ACSL4 cells

Despite evidence linking the action of ACSL4 to the development of various types of cancer including colon, hepatocellular carcinoma, prostate and breast cancer, very little is known regarding the signal transduction mechanism by which ACSL4 influences these lesions.

**Figure 1:**
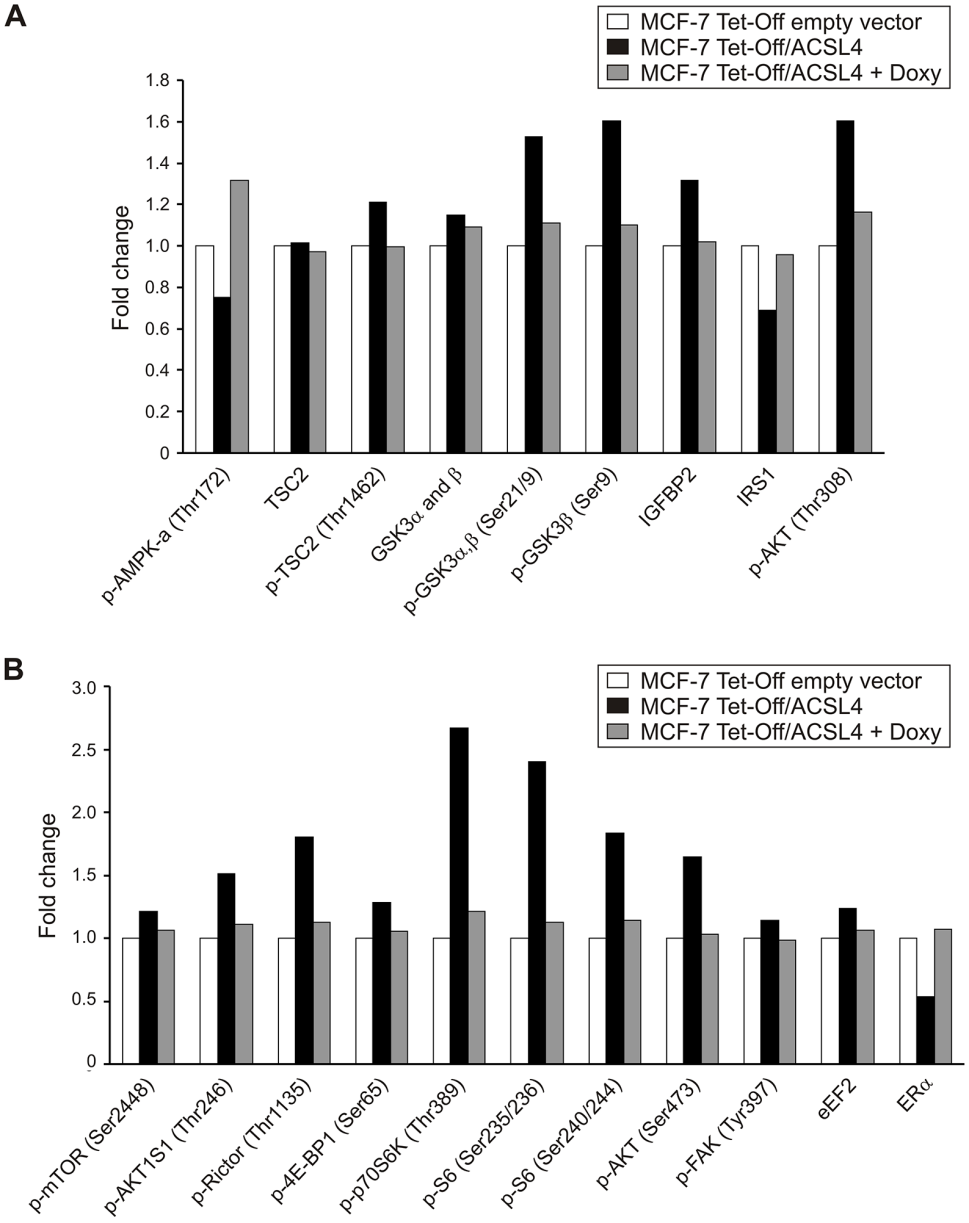
Identification of modified protein expression or phosphorylation of the mTOR pathway in ACSL4-overexpressing cells using RPPA Proteins were extracted from MCF-7 Tet-Off empty vector, MCF-7 Tet-Off/ACSL4 cells and doxycycline-treated MCF-7 Tet-Off/ACSL4 (Doxy, 1 ug/ml, 48 h) cells and subjected to RPPA. Protein expression levels are presented as fold changes in MCF-7 Tet-Off/ACSL4 cells and doxycycline-treated MCF-7 Tet-Off/ACSL4, both compared to MCF-7 Tet-Off empty vector cells. Data refer to upstream regulators **A.** and components and substrates **B.** of the mTOR pathway.

In order to study the signaling pathways triggered by ACSL4, we first defined a functional protein signature of the ACSL4 pathway by using the reverse phase protein array (RPPA), a high-throughput antibody-based technique developed for functional proteomic studies to measure phosphorylation states, as well as total levels of key signaling pathway intermediaries. The analysis was performed on lysates derived from MCF-7 cells stably transfected with ACSL4 using the tetracycline Tet-Off system (MCF-7 Tet-Off/ACSL4), MCF-7 Tet-Off empty vector and doxycycline-treated MCF-7 Tet-Off/ACSL4 cells, the latter used to specifically override ACSL4 expression. The pattern of protein expression and/or phosphorylation was remarkably different between MCF-7 Tet-Off/ACSL4 and MCF-7 Tet-Off empty vector (Figure 1).

A functional annotation analysis using the bioinformatic program DAVID [11] on the basis of RPPA data is shown in Table 1. This analysis revealed that mTOR signaling pathway was the second lowest *p*-value. Together with insulin and ERbB signaling and cancer, they were the top four signaling pathways with the lowest *p*-values. Pathways in cancer found included prostate cancer, chronic and acute myeloid leukemia, pancreatic, and colorectal cancer, glioma, melanoma, endometrial and non small cell lung cancer. The p53 signaling pathway was also included.

**Table 1:** Functional annotation analysis of RPPA data from ACSL4-overexpressing MCF-7 cells using the bioinformatic program DAVID

Term	Genes	%	*p*-value
Insulin signaling pathway	15	30	5.7E-13
mTOR signaling pathway	10	20	1.3E-10
ErbB signaling pathway	11	22	7.1E-10
Pathways in cancer	17	24	9.5E-10
Prostate cancer	10	20	1.8E-8
Adipocytokine signaling pathway	9	18	3.3E-8
Chronic myeloid leukemia	9	18	8.1E-8
Acute myeloid leukemia	8	16	2.4E-7
Pancreatic cancer	8	16	1.1E-6
Small cell lung cancer	8	16	3.1E-6
Colorectal cancer	8	16	3.1E-6
Neurotrophin signaling pathway	9	18	4.0E-6
Glioma	7	14	7.8E-6
p53 signaling pathway	7	14	1.2E-5
Apoptosis	7	14	5.1E-5
Focal adhesion	9	18	1.3E-4
Melanoma	6	12	2.1E-4
Cell cycle	7	14	3.8E-4
Chemokine signaling pathway	8	16	5.4E-4
Endometrial cancer	5	10	6.8E-4
Non-small cell lung cancer	5	10	7.8E-4

Enriched terms of KEGG_PATHWAY database were used for the analysis. The *p*-value is calculated as modified Fisher Exact *p*-value for gene enrichment analysis. It ranges from 0 to 1, with Fisher Exact *p*-value = 0 representing perfect enrichment. Usually *p*-values equal to or lower than 0.05 are considered strongly enriched in the annotation categories. Percentages refer to number of genes involved over total of genes in the term.

RPPA data showed that ACSL4 expression enhanced the phosphorylation of mTOR on Ser2448 without changes in its protein levels. mTOR has emerged as a critical effector in cell signaling pathways commonly dysregulated in human cancer. mTOR is regulated by growth factors and nutrients, which indicates that it is at the interface of two different growth signals [12], and comprises a rapamycin- and nutrient-sensitive multiprotein complex (mTORC1) and a growth factor-sensitive nutrient-insensitive complex (mTORC2). mTORC1 modulates at least two separate downstream pathways that are conjectured to control the translation of a specific subset of mRNAs [13, 14]. ACSL4 expression enhanced the phosphorylation of p70S6K on Thr389, a downstream target of mTORC1, and its substrate, S6 protein on Ser235/236 and 240/244 (Figure 1). The phosphorylation of the initiation factor 4E-BP1 on Ser65 was also enhanced, increasing the availability of functional eukaryotic initiation factor 4E (eIF4E). An increase was also observed in the phosphorylation of Rictor on Thr1135, a subunit of mTORC2 complex, which is in line with evidence showing that the activation of mTORC2 by growth factor signaling is linked to the specific phosphorylation of its Rictor component on Thr1135 [15].

Another interesting phosphoprotein that is modulated by ACSL4 expression is the protein encoded by gene PRKAA1 (AMPK or protein kinase, AMP-activated, alpha 1 catalytic subunit). When phosphorylated, this protein negatively regulates the mTORC1 complex by phosphorylating its Raptor (regulatory-associated protein of mTOR) component and phosphorylating and activating the tuberous sclerosis complex 2 (TSC2) [16].

TSC2 (also known as tuberin) forms a heterodimer with TSC1 (also known as hamartin), which negatively regulates mTORC1 signaling [12, 17]. ACSL4 expression reduces the phosphorylation levels of TSC2 on Thr172, decreasing its activity and inactivating the TCS1/2 complex. The phosphorylation and the inhibition of TSC2 by AKT is the earliest link between mTORC1 and a pathway dysregulated in cancer [12]. This observation reinforce the participation of mTORC1 in ACSL4 signature. In addition, we observed an increase in the phosphorylation of AKT on Ser308 and 473, and AKTS1 (AKT1 Substrate 1, Proline-Rich/PRAS40) on Thr246. The mTORC2 complex phosphorylates AKT on Ser473, and these results add up to the well-known fact that the activation of AKT places mTOR on both sides of AKT signaling [18].

GSK3α and GSK3β are critical negative regulators of diverse signaling pathways, including mTOR [19]. These are two additional phosphoproteins whose levels exhibit an important increase in response to ACSL4 expression, and whose phosphorylation on Ser21 and Ser9, respectively, inhibits GSK3 activity and therefore contributes to mTOR activation [20, 21]. ACSL4 expression also stimulates the protein levels of growth factors and their receptors, such as the insulin-like growth factor binding protein (IGFBP2), also an upstream regulator of mTOR. ACSL4 expression decreases the protein levels of insulin receptor substrate 1 (IRS1), whose recruitment in insulin pathway can activate mTORC1 through AKT activation. In turn, the activation of p70S6K by mTORC1 promotes the phosphorylation of IRS1 and reduces its stability, an auto-regulatory pathway or negative feedback loop that has been shown to have profound implications for both metabolic diseases and tumorigenesis [22, 23].

It is known that both in normal and transformed cells the focal adhesion kinase (FAK) increases cell motility. This effect is activated by FAK phosphorylation on Tyr397 [24], an event in turn enhanced by ACSL4 expression. FAK protein expression is elevated in many highly malignant human cancer types, and studies have shown that FAK signaling can promote changes in cell shape and the formation of podosomes or invadopodia, which in turn leads to an invasive cell phenotype [24]. GSK3β has also been implicated in the negative regulation of FAK activity [24].

**Figure 2:**
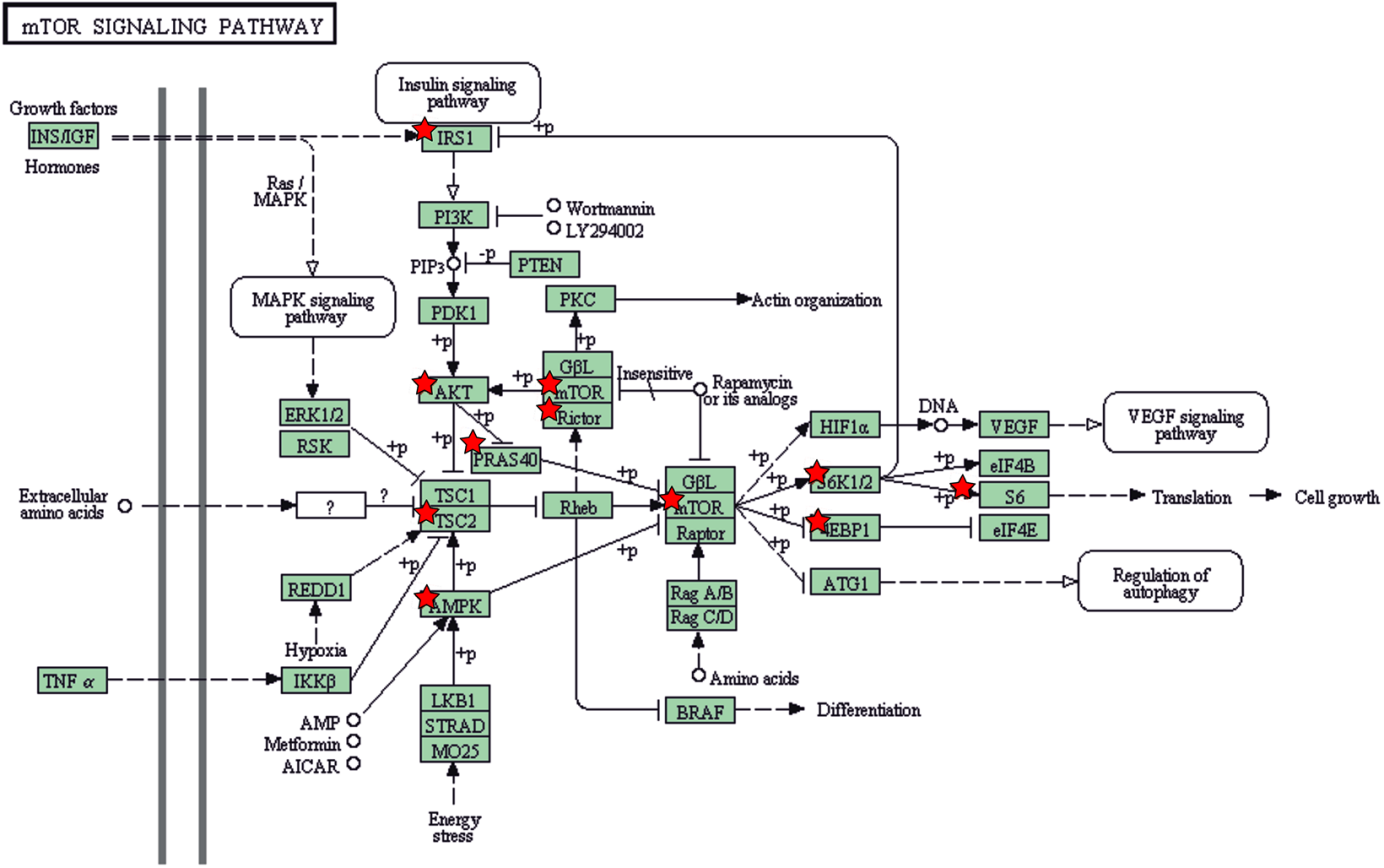
Description of ACSL4 effects on mTOR signaling pathway components The mTOR pathway scheme was obtained from KEGG_PATHWAY database. Analysis was performed by means of the DAVID bioinformatic tool on the basis of RPPA data. Red stars highlight ACSL4-regulated proteins in RPPA.

The specificity of ACSL4 was established by the specific inhibition of its expression in doxycycline-treated MCF-7 Tet-Off/ACSL4 cells. In this case, RPPA data showed a pattern similar to that of MCF-7 Tet-Off empty vector, further supporting the role of ACSL4 in the effects observed. All these findings suggest that the mTOR pathway is part of the signal transduction involved in ACSL4 effects. In addition, RPPA analyses confirmed previous findings showing that ACSL4 negatively regulates ERα protein expression (Figure 1). This effect was reversed by doxycycline treatment, which again shows the specificity of the effects triggered by ACSL4 and the inverse relationship between ACSL4 and ERα. A KEGG pathway map scheme obtained from DAVID analysis [11] using the RPPA data shows ACSL4- regulated proteins involved in the mTOR pathway (Figure 2).

### Identification of ACSL4-responsive genes in MCF-7 Tet-Off/ACSL4 cells through transcriptome analysis

We next focused on the identification of ACSL4-responsive genes in MCF-7 Tet-Off/ACSL4 cells compared with MCF-7 Tet-Off empty vector cells and only considered loci rendering a log_2_ (fold change) > 2 between MCF-7 Tet-Off/ACSL4 and MCF-7 Tet-Off empty vector. Out of 32247 successfully sequenced loci, 3944 were significantly and differentially expressed in MCF-7 Tet-Off/ACSL4 samples. Among them, 2501 were upregulated and 1443 were downregulated. Using Ingenuity Pathway Analysis (IPA) [25], enriched functional categories of gene networks were ranked relating to the transcripts regulated in ACSL4-responsive gene sets acquired from the RNA-Seq data. When ACSL4 was expressed, analyses showed cancer to be the disease with the lowest *p*-value among diseases and disorders. The top three biofunctions which were IPA-predicted to be increased in RNA-Seq data were cell movement, migration and proliferation. Enriched canonical pathways were also analyzed through IPA, which interestingly revealed p70S6K, mTOR and the signaling of molecular mechanisms of cancer to be among the top canonical pathways triggered by ACSL4 with the lowest *p*-values (Table 2).

**Table 2:** Enriched canonical pathways in ACSL4-overexpressing MCF-7 cells

Ingenuity Canonical Pathways	−log(*p*-value)
EIF2 Signaling	21.2
Protein Ubiquitination Pathway	14.6
Regulation of eIF4 and p70S6K Signaling	8.88
Molecular Mechanisms of Cancer	8.26
mTOR Signaling	8.18
Mitochondrial Dysfunction	7.47
NRF2-mediated Oxidative Stress Response	6.88
Cell Cycle: G1/S Checkpoint Regulation	6.81
ATM Signaling	6.81
Hypoxia Signaling in the Cardiovascular System	6.67
Oxidative Phosphorylation	6.25
PI3K/AKT Signaling	6.19
14–3-3-mediated Signaling	5.72
NGF Signaling	5.50
PTEN Signaling	5.14
Antiproliferative Role of TOB in T Cell Signaling	5.12
Pancreatic Adenocarcinoma Signaling	4.94
D-myo-inositol (1,3,4)-trisphosphate Biosynthesis	4.76
ERK5 Signaling	4.66
Type II Diabetes Mellitus Signaling	4.66
Hereditary Breast Cancer Signaling	4.55
TGF-β Signaling	4.55

IPA analysis of RNA-Seq data towards canonical pathways. Pathways are shown which were designated significantly enriched by the software, with a *p*-value < 0.05 by Fisher's Exact test.

In other words, the ingenuity canonical pathways obtained with the ACSL4 transcriptome signature correlate with the ACSL4-functional proteomic signature, where the AKT-mTOR-p70S6K signaling pathway seems to be one of the most important ACSL4 signatures.

Supplementary Tables S1 and S2 also show ACSL4-regulated genes related to the mTOR pathway and eIF4 and p70S6K signaling, respectively. An important number of well-known genes were observed which are involved in mTOR signaling and regulate cell growth by controlling mRNA translation, ribosome biogenesis, autophagy and metabolism. In addition, results from RNA-Seq showed that ACSL4 overexpression causes a strong reduction in the expression of WIF1. The protein encoded by this gene inhibits WNT (Wingless-Type MMTV Integration Site Family) proteins, which are extracellular signaling molecules playing a role in embryonic development. In particular, ACSL4 increased the expression of WNT6 and WNT10A.

In summary, these results further suggest that the mTOR signaling pathway, as well as upstream and downstream regulators of mTOR, are part of the targets of ACSL4 effects.

### Lipoxygenase metabolites mediation of ACSL4 effects on the mTOR pathway

**Figure 3:**
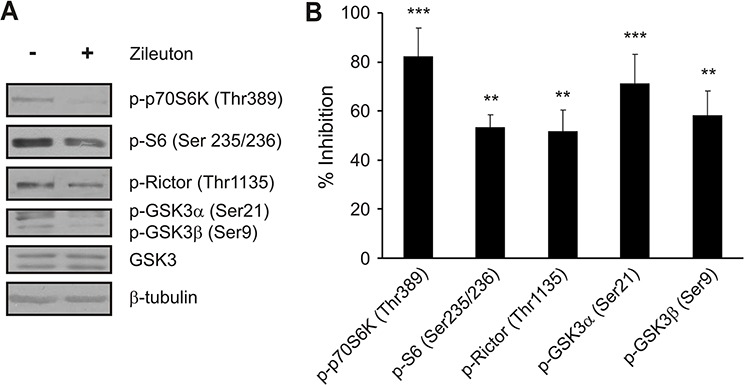
Role of LOX-5 metabolites in ACSL4 effects on the mTOR pathway MCF-7 Tet-Off/ACSL4 cells were incubated in the presence or absence of zileuton (LOX-5 inhibitor, 500 μM) for 24 h. Whole cell extracts were obtained as described in Materials and Methods. Western blot was performed using the indicated antibodies. Representative blots **A.** and integrated optical density of protein levels **B.** quantified and normalized with the corresponding β-tubulin signal. Data represent the means of percentages of inhibition ± SD of three independent experiments. ***p < 0.001 and **p < 0.01 vs. control cells.

We have previously reported the involvement of AA lipoxygenase-5 (LOX-5) metabolites in the action of ACSL4 on cell proliferation and tumor growth [4, 6]. Considering the results showed above indicating that ACSL4 might be a novel regulator of mTOR, we next validated these results evaluating whether these metabolites are involved in ACSL4 effects on mTOR signaling. For this purpose, we used zileuton to inhibit LOX-5 as previously described [4, 6]. MCF-7 Tet-Off/ACSL4 cells were treated as shown in Materials and Methods and cell lysates were analyzed by Western blot using p70S6K (Thr389), S6 (Ser235/236), Rictor (Thr1135), GSK3αβ (Ser21/9) and GSK3 antibodies as reference for the mTOR signal. LOX-5 inhibition reduced the phosphorylation of p70S6K and Rictor (Figure 3) and inhibited cell proliferation (data not shown) as previously described [4, 6], demonstrating that lipoxygenase metabolites are in part the mediators of ACSL4 effects on the regulation of the mTOR pathway.

### Validation of ACSL4 effects on the mTOR pathway

Considering the results shown above indicating that ACSL4 might be a novel regulator of mTOR, we further analyzed the mTOR pathway by Western blot using the same cell model used in the RPPA analysis and two additional breast cancer cell models. We measured the basal expression level of some of the mTOR signaling and related molecules, along with the effect of the up and downregulation of ACSL4 expression.

**Figure 4:**
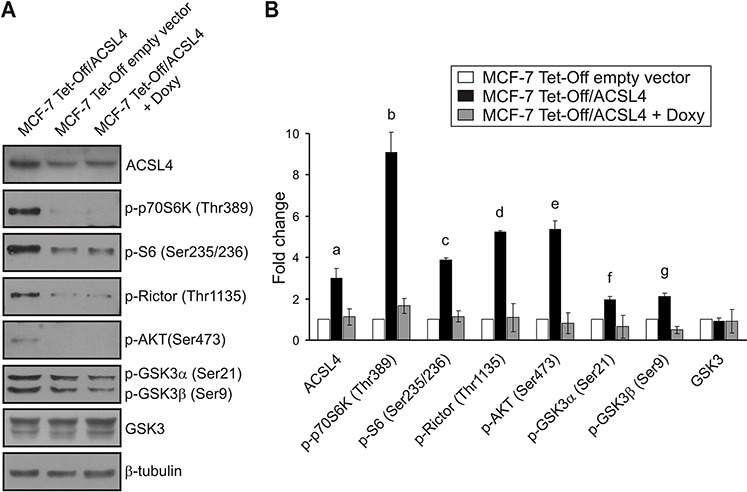
Validation of ACSL4 effects on the mTOR pathway in MCF-7 cells Whole cell extracts were obtained as described for RPPA from MCF-7 Tet-Off empty vector, MCF-7 Tet-Off/ACSL4 and doxycycline-treated MCF-7 Tet-Off/ACSL4 cells (Doxy, 1 ug/ml, 48 h). Western blot was performed using the indicated antibodies. Representative blots **A.** and integrated optical density of protein levels **B.** quantified and normalized with the corresponding β-tubulin signal. As shown in Figure 1, data represent the means of fold changes ± SD of three independent experiments. a, b, c, d and e: ***p < 0.001; f and g: *p < 0.05 vs. MCF-7 Tet-Off empty vector cells.

As shown in Figure 4, ACSL4 protein levels were significantly upregulated in the MCF-7 Tet-Off/ACSL4 cells, as compared to MCF-7 Tet-Off empty vector cells, and returned to basal levels in the lyses obtained from doxycycline-treated MCF-7 Tet-Off/ACSL4 cells, confirming that this model is useful to analyze the role of ACSL4. A significant increase in the phosphorylation of p70S6K on Thr389 and its substrate, ribosomal protein S6, on Ser235/236 showed that mTORC1 is activated by ACSL4 expression. An increase was also observed in the phosphorylation of Rictor on Thr1135, substrate of p70S6K and component of mTORC2 complex. In addition, the enhancement detected in AKT phosphorylation on Ser473 indicates that its kinase, mTORC2, is also activated by ACSL4 expression. GSK3α and GSK3β phosphorylation levels on Ser21/9, and not total GSK3 levels, were increased in response to ACSL4 expression; GSK3 activity was thus inhibited and contributed to mTOR activation.

These results confirmed those obtained by RPPA. As expected, the doxycycline treatment of MCF-7 Tet-Off/ACSL4 cells resulted in the inhibition of the effect observed with the overexpression of ACSL4 (Figure 4).

### Effects of ACSL4 expression on the mTOR pathway in T47D cells

**Figure 5:**
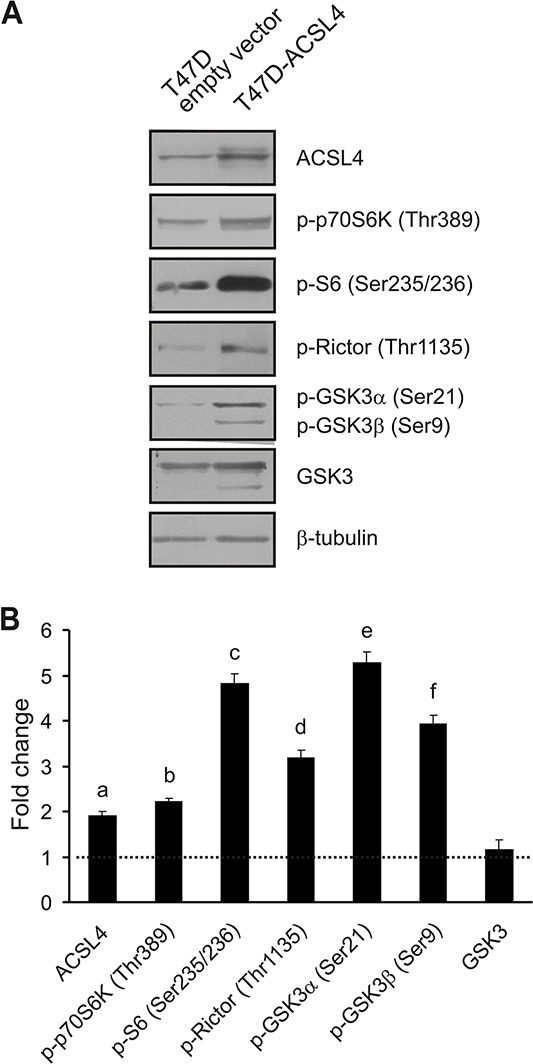
ACSL4 effects on the mTOR pathway in T47D cells T47D cells were transfected with the plasmid containing ACSL4 cDNA (T47D-ACSL4) or empty plasmid (T47D empty vector). Forty-eight hours post transfection, cells were selected with G418 (500 μg/ml) antibiotic. One month post selection, whole cell extracts were obtained and analyzed by Western blot using the indicated antibodies. Representative blots **A.** and integrated optical density of protein levels **B.** quantified and normalized with the corresponding β-tubulin signal. Data represent the means of fold changes (T47D-ACSL4 vs. T47D empty vector) ± SD of three independent experiments. Dotted line indicates control values. a, b, c, d, e and f: ***p < 0.001 vs. T47D empty vector cells.

In order to further evaluate the effect of ACSL4 expression on the mTOR pathway observed with the MCF-7 Tet-Off/ACSL4 cells, we next produced an ACSL4-overexpressing system using a different non-aggressive breast cancer cell line, i.e. T47D cells. (T47D ACSL4) (Figure 5). The basal expression levels of some of the mTOR signaling and related molecules were measured by Western blot analysis, together with the effect of ACSL4 upregulation. Consistent with the results described above, the phosphorylation pattern of mTOR related to phosphoprotein levels observed in T47D-ACSL4 cells was the same as that observed in MCF-7 Tet-Off/ACSL4 cells.

### Effects of ACSL4 knockdown on the mTOR pathway in MDA-MB-231 cells

**Figure 6:**
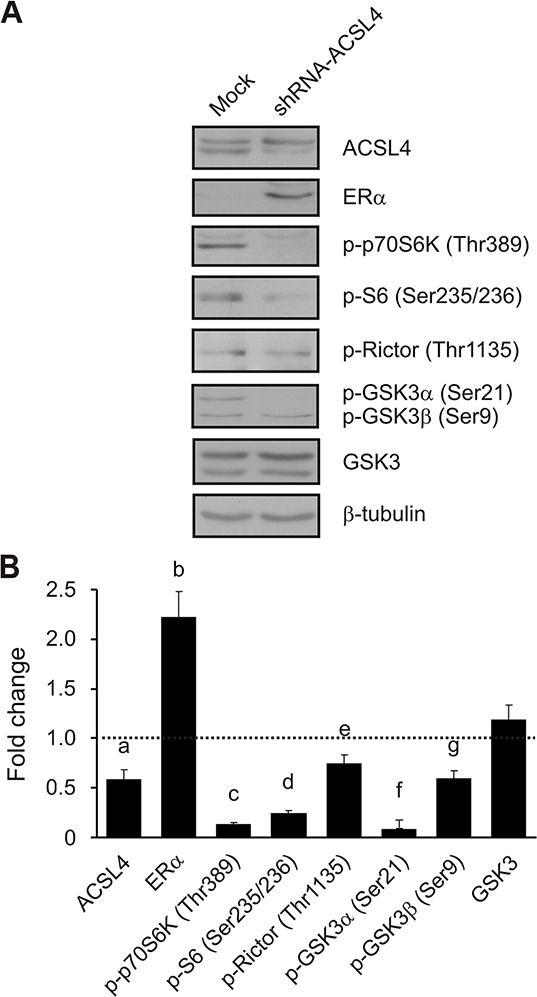
ACSL4 effects on the mTOR pathway in MDA-MB-231 cells MDA-MB-231 cells were transfected with the pSUPER.retro plasmid containing a shRNA targeted to ACSL4 (shRNA-ACSL4) or an empty plasmid (mock). Forty-eight hours post transfection, cells were selected with puromycin (1 μg/ml) antibiotic. One month post selection, whole cell extracts were obtained and analyzed by Western blot as previously described using the indicated antibodies. Representative blots **A.** and integrated optical density of protein levels **B.** quantified and normalized with the corresponding β-tubulin signal. Data represent the means of fold changes (shRNA-ACSL4 vs. mock) ± SD of three independent experiments. Dotted line indicates control values. a, b, c, d, f and g: ***p < 0.001; e: **p < 0.01 vs. mock transfected cells.

Given that overexpression experiments could force ACSL4 action, and to further validate the results described above through a control experiment, we next disrupted the expression of endogenous ACSL4 using small hairpin RNA (shRNA) in the highly aggressive MD-MB-231 breast cancer cells, which constitutively overexpress ACSL4. Western blot analyses showed that the shRNA targeting ACSL4 markedly decreased the expression of ACSL4 protein approximately by 40% (Figure 6). ACSL4 knockdown markedly decreased the protein levels of p70S6K (Thr389) and its substrates, S6 (Ser235/236) and Rictor (Thr1135), indicating that mTORC1 activity was reduced. GSK3α and β phosphorylation levels (Ser21/9), and not total GSK3 levels, were decreased in response to ACSL4 knockdown, indicating that GSK3 was active and thus contributed to mTOR inhibition. Concomitantly with the reduction observed in the mTOR signal, there was a significant reduction in cell proliferation, invasion and migration (data not shown), as previously described [6]. Remarkably, ACSL4 knockdown increased ERα expression, confirming the regulation of ER protein expression by ACSL4 (Figure 6).

### Inhibition of cell proliferation through the combination of sub-effective doses of ACSL4 and mTOR inhibitors

Rapamycin, a macrolide fungicide, has generated significant interest as a potential anticancer drug. Rapamycin inhibits mTOR by binding to one of the members of the immunophilin family of FK 506-binding proteins (FKBP12) [26, 27].

**Figure 7:**
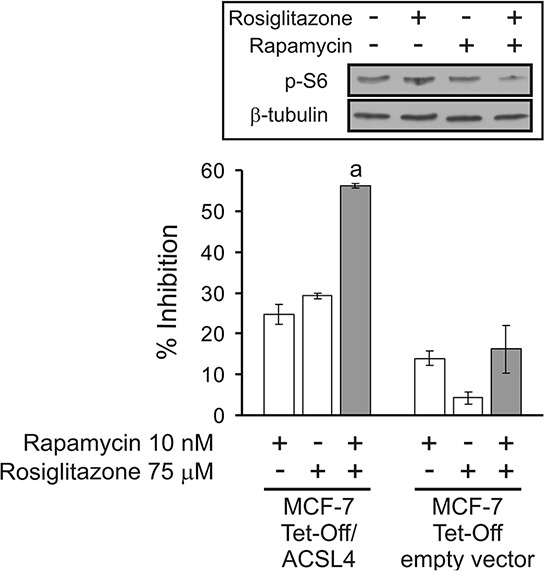
Cell proliferation inhibition by combining sub-effective doses of ACSL4 and mTOR pathway inhibitors MCF-7 Tet-Off empty vector and MCF-7 Tet-Off/ACSL4 cells were plated at a density of 4000 cells/well in 96-well plates with 10% FBS-supplemented D-MEM medium and allowed to adhere overnight at 37°C in a humidified 5% CO_2_ atmosphere. The medium was then changed to serum-free medium. After 24 h, the cells were switched to 10% FBS-supplemented D-MEM medium with rapamycin (10 nM) and/or rosiglitazone (75 μM) for 96 h. Subsequently, cell proliferation was measured by BrdU incorporation assays as described before [6]. Data are presented as inhibition of cell proliferation compared to control cells. White bars indicate a single inhibitor treatment while grey bars indicate combined inhibitor treatment. Data are presented as means ± SD. a: ***p < 0.001 vs. single inhibitors. **Inset**: MCF-7 Tet-Off/ACSL4 cells were incubated in the presence or absence of rosiglitazone (75 μM) alone or in combination with rapamycin (10 nM) for 48 h. p-S6 protein levels were evaluated by Western blot and a representative blot is shown.

In this context, and in order to validate a potential clinical significance of the novel effect of ACSL4 on mTOR, we used a combined pharmacological approach involving rapamycin in combination with rosiglitazone, an inhibitor of ACSL4 activity [28, 29], to elucidate the signaling mechanism underlying ACSL4 impact on cell proliferation. To examine the sensitivity of MCF-7 Tet-Off/ACSL4 cells to rapamycin, cells were treated at different concentrations (1–1000 nM) for 4 days and cell proliferation was measured by BrdU (5-bromo-2′-deoxyuridine) incorporation and MTT (3-(4,5-dimethyl-2-thiazolyl)-2,5-diphenyl-2H-tetrazoliumbromide) assays as described in Materials and Methods. The minimal dose of rapamycin that produced a significant inhibition was 10 nM, while, as previously described, the minimal dose of rosiglitazone to produce a significant inhibition was 75 μM. Therefore, we used these submaximal doses of the inhibitors in order to observe the efficacy of this combination of drugs. Figure 7 describes the results obtained using BrdU incorporation, since both MTT and BrdU assays showed the same results. Treatment with 10 nM rapamycin or 75 μM rosiglitazone produced a significant inhibition on MCF-7 Tet-Off/ACSL4 cell proliferation. Interestingly, the combination of these drugs enhanced the inhibition of cell proliferation, suggesting the potential for additive, synergistic inhibitory effects. Moreover, the same synergistic effect was observed for inhibitor combination on p-S6 protein levels, a representative component of mTOR signaling (Figure 7, inset).

The same approach used to treat MCF-7 Tet-Off empty vector cells revealed low but significant inhibition produced by rapamycin treatment but no combined inhibitory effect of these drugs. A possible explanation for this finding may involve the low expression of ACSL4 in these cells and, therefore, little contribution of ACSL4 to mTOR activity.

When the effect of these drugs was analyzed in the highly aggressive MDA-MB-231 breast cancer cells, which constitutively overexpress ACSL4, no effects were observed for rapamycin at any concentrations (data not shown). The lack of response to rapamycin treatment of MDA-MB-231 cells has been previously described by other authors [30]. As an explanation for this finding, mTOR may potentially activate cell growth in very aggressive cells by mechanisms other than its well-known nutrient-sensitive multiprotein complex. In turn, sensitivity to rosiglitazone could be explained by the fact that MDA-MB-231 breast cancer cells express very high levels of ACSL4 and, as shown in the results above, ACSL4 affects the up and downregulators of mTOR and regulates mTORC1 and mTORC2 activities.

### Inhibition of cell proliferation through the combination of sub-effective doses of rosiglitazone and 4-hidroxitamoxifen (4-OHTAM)

We have shown that knocking down ACSL4 expression in the aggressive triple negative breast cancer cell line MDA-MB-231 induces ERα expression (Figure 6). As expected, and in agreement with previous results [4], ACSL4 overexpression decreased the level of ERα (see Figure 1). In our interpretation of these data, the inhibition of ACSL4 might force the tumor to restore the estrogen signaling pathway for continuous growth and hormonal sensitivity, which is why the blockade of both the ACSL4 and estrogen pathways together might leave the tumor with extremely few options.

**Figure 8:**
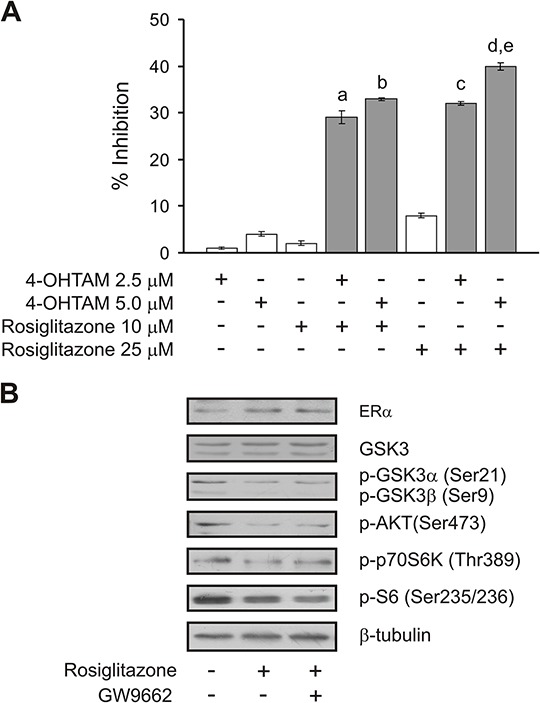
Cell proliferation inhibition by combining sub-effective doses of ACSL4 and ER pathway inhibitors MCF-7 Tet-Off/ACSL4 cells were plated as described in Figure 7 and then incubated with rosiglitazone (10 or 25 μM) and/or 4-hidroxitamoxifen (4-OHTAM 2.5 or 5 μM) for 96 h. Subsequently, cell proliferation was measured by BrdU incorporation assays **A.**. Data are presented as inhibition of cell proliferation compared to control cells. White bars indicate a single inhibitor treatment while grey bars indicate combined inhibitor treatment. Data are presented as means ± SD. a, b, c, and d: ***p < 0.001 vs. corresponding single inhibitors; e: ***p < 0.001 vs. 4-OHTAM 2.5 μM + rosiglitazone 10 μM. MCF-7 Tet-Off/ACSL4 cells were incubated in the presence or absence of rosiglitazone (75 μM) alone or in combination with GW9662 (10 μM) for 24 h. ERα and mTOR-related protein levels were evaluated by Western blot and a representative blot is shown **B.**.

By means of the MCF-7 Tet-Off/ACSL4 model system, which involves a reduction in ERα levels, we used a pharmacological approach to inhibit cell proliferation through a combination of sub-maximal doses of tamoxifen (4-OHTAM) and rosiglitazone. As shown in Figure 8A, treatment with the inhibitors alone did not produce a significant inhibition in cell proliferation. However, the combination of the two inhibitors was much more efficient in inhibiting cell proliferation than 4-OHTAM or rosiglitazone individually, showing a remarkable synergistic effect. These results open up the possibility for inhibitor combinations which might prevent the loss of hormonal response in tumors that begin to overexpress ACSL4 and begin to reduce the levels of ER. Levels of ERα, p-S6, p-GSK3αβ, p-AKT (Ser473) and p-p70S6K were monitored to confirm that rosiglitazone treatment indeed increased ERα expression and decreased the mTOR signal as expected (Figure 8B). These results are in agreement with previous results showing that mTOR inhibition by rapamycin reverses acquired endocrine therapy resistance of breast cancer cell and cell proliferation [31].

**Figure 9:**
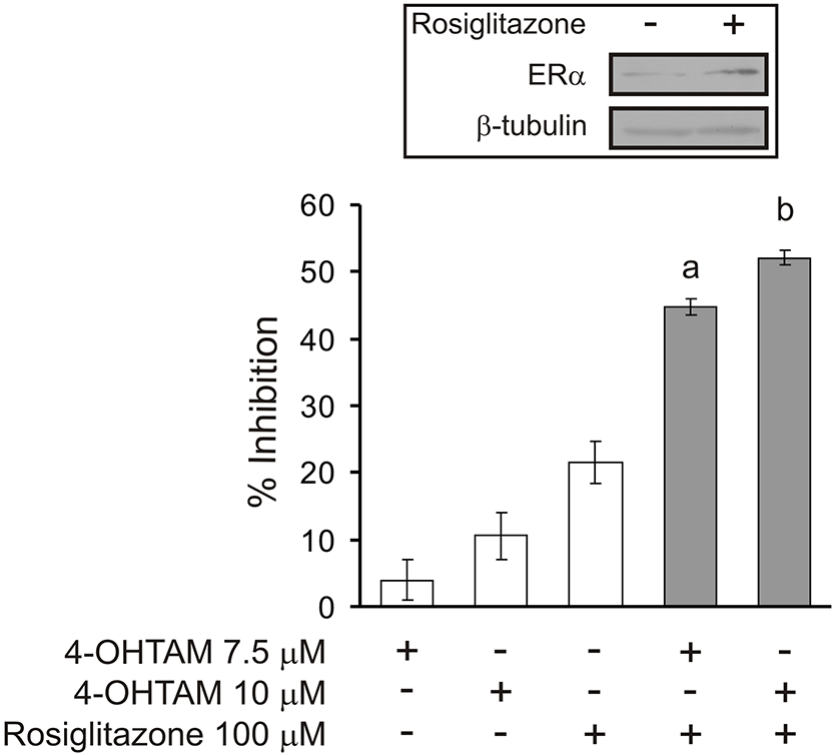
Cell proliferation inhibition by combining sub-effective doses of ACSL4 and ER pathway inhibitors in MDA MB-231 cells MDA-MB-231 cells were plated as described for MCF-7 Tet-Off/ACSL4 cells in Figure 7 and then incubated with rosiglitazone (100 μM) and/or 4-hidroxitamoxifen (4-OHTAM 7.5 or 10 μM) for 96 h. Subsequently, cell proliferation was measured by BrdU incorporation assays. Data are presented as inhibition of cell proliferation compared to control cells. White bars indicate a single inhibitor treatment while grey bars indicate combined inhibitor treatment. Data are presented as means ± SD. a and b: ***p < 0.001 vs. corresponding single inhibitors. **Inset**: MDA-MB-231 cells were incubated in the presence or absence of rosiglitazone (100 μM) for 48 h. ERα protein levels were evaluated by Western blot and a representative blot is shown.

Since, as described above, the triple negative MDA-MB-231 breast cancer cells are sensitive to rosiglitazone, we also studied whether the inhibition of ACSL4 activity could reverse ER therapy resistance in cells that do not express ER. Figure 9 shows the effect of a combination of rosiglitazone and 4-OHTAM on cell proliferation. The combination of the two inhibitors was much more efficient in inhibiting cell proliferation than 4-OHTAM or rosiglitazone individually. Figure 9 inset shows again that rosiglitazone treatment indeed increased ERα expression, as shown in Figure 8B. Again, these results suggest promising inhibitor combinations which might prevent the loss of hormonal response in triple negative tumors overexpressing ACSL4.

### *In vivo* therapy of solid tumors in mice

On the basis of our previous *in vitro* results and the demonstration that modulating ACSL4 inhibition results in the upregulation of ER with a consequent change in cell phenotype and the sensitivity of tamoxifen, the logical next step was to analyze the effect of ACSL4 inhibitor and tamoxifen on tumor growth *in vivo*.

The MDA-MB-231 cell line is known to form tumors with a triple negative signature that do not respond to hormone treatment; therefore, the MDA-MB-231 xenograft model was a very good challenge to demonstrate that ACSL4 inhibitor and ER inhibitor are working in a concerted manner and demonstrate a synergistic effect of the inhibitors as a potential therapeutic protocol.

For MDA-MB-231 tumor xenografts, four days after cell injection the tumor-bearing mice were randomized into the following four groups (five animals per group) and received intraperitoneal injections and oral administration of the respective drugs for 25 consecutive days:

Group 1 (MDA-MB-231 cell xenografts treated with vehicle), Group 2 (MDA-MB-231 cell xenografts treated with tamoxifen), Group 3 (MDA-MB- 231 cell xenografts treated with rosiglitazone), Group 4 (MDA-MB-231 cell xenografts treated with a combination of the two drugs at the same doses used for the individual injections).

**Figure 10:**
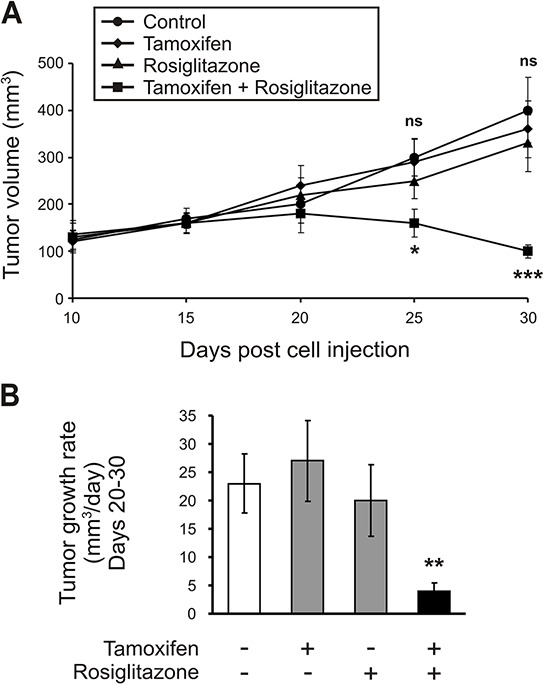
Effect of ACSL4 and ER pathway inhibitors in the MDA-MB-231 human breast xenograft model Mice bearing MDA-MB-231 tumor xenografts were treated with either vehicle (control), rosiglitazone or tamoxifen alone, or with a combination of the two inhibitors at doses described in Materials and Methods for 26 consecutive days. Comparison of average tumor volume **A.** and tumor growth rate between days 20–30 **B.** was determined. Data are presented as means ± SD, *n* = 5. Asterisks indicate significant differences between tumor volumes by two-way ANOVA (A) and between tumor growth rates by one-way ANOVA (B). ns vs. single inhibitors; *p < 0.05, **p < 0.01, ***p < 0.001 vs. corresponding single inhibitors.

Although the MDA-MB-231 xenograft growth rate varies among studies reported in the literature, our tumor xenografts were in the range of those previously reported [24]. The average animal body weight was 23.5 g at the beginning of the treatment and no significant differences in body weight were observed between the different treatment groups at the end of the experiment. The amount of food intake in the control compared to the treated groups was not significantly different throughout the experiment. However, there was a significant inhibition in average tumor volume and growth rate in animals subjected to combination therapy compared to those that received single drug-based treatments or drug vehicle after injection of MDA-MB-231 cells (Figure 10A and 10B, respectively). The tumors from mice treated with drug combination were clearly smaller than those from either the control group or single drug treatment groups.

## DISCUSSION

This study was undertaken to elucidate the signal transduction involved in the effects of ACSL4 on cell growth. Even though it was previously demonstrated that this enzyme plays a causal role in the control of breast cancer aggressiveness, the mechanism underlying this process has yet to be fully elucidated.

In the present work we demonstrate that ACSL4 can be considered a novel activator of the mTOR pathway, for both mTORC1 and mTORC2 components, and that ACSL4 expression can trigger several different mechanisms to regulate mTOR.

The specificity of ACSL4 action on mTOR signaling was assessed by the specific inhibition of ACSL4 expression by doxycycline in the MCF-7 Tet-Off/ACSL4 model, by the expression of ACSL4 in the non-aggressive T47D breast cancer cell line and by the downregulation of ACSL4 expression in the very aggressive MDA-MB-231 breast cancer cells that constitutively expresses ACSL4. mTOR comprises a rapamycin- and nutrient-sensitive multiprotein complex (mTORC1) and a growth factor-sensitive nutrient-insensitive complex (mTORC2). A direct regulation of mTOR activity by ACSL4 is demonstrated by the fact that ACSL4 expression increases the phosphorylation of mTOR on Ser2448.

A major leap forward in understanding mTORC1 regulation was the discovery that TSC1/2 bipartite protein complex negatively controls its activity [12, 32]. Mutations in either the *tsc1* or the *tsc2* genes cause the hamartomatous syndrome tuberous sclerosis complex (TSC) [32]. The discovery of the connection between TSC and mTORC1 pathway provided the first molecular link between mTOR and cancer. ACSL4 contributes to the regulation of the TSC1/2 complex is through the decrease in the phosphorylation levels of AMPK phosphoprotein on Thr172 decreasing its activity and inactivating the TCS2 complex. As mentioned before, this phosphoprotein negatively regulates the mTORC1 complex by phosphorylating its Raptor component and phosphorylating and activating TCS2 [12]. These results strongly suggest that ACSL4 expression modulates at least two separate signaling pathways which are conjectured to control mTOR activity.

An additional mechanism through which ACSL4 regulates mTOR activity is the increase in the phosphorylation of AKT1S1. AKT participates in mTORC1 activation in an independent fashion by phosphorylating AKT1S1, causing its dissociation from Raptor and suppressing mTORC1 inhibition.

Diverse signals regulate TSC1/2, which suggests that, like mTORC1, TSC1/2 is a signal integration center. Positive growth signal from RAS-MAPK pathway inhibits TSC2, although the phosphorylation and inhibition of TSC2 by AKT are the clearest links between mTORC1 and pathway deregulation in cancer [12, 18].

In contrast to growth-factor-driven activation of mTORC1, hypoxia, AMPK activation resulting from depletion of cellular energy, Wnt-GSK3 signaling and glucocorticoids all inhibit mTORC1 by promoting TSC1/2 activation [19, 33, 34]. The phosphorylation of GSK3 is a potent inhibitor of its activity and our results clearly show that ACSL4 expression increase the phosphorylation of GSK3αβ. As GSK3 has been shown to inhibit the Wnt signaling pathway, the inhibition of GSK3 activity by phosphorylation suggests that Wnt signaling is part of the mechanism of action of ACSL4 expression.

The aberrant regulation of the Wnt signaling pathway is a prevalent theme in cancer. From early observations that WNT overexpression could lead to malignant transformation of mouse mammary tissue to the most recent genetic discoveries gleaned from tumor genome sequencing, the Wnt pathway continues to evolve as a central mechanism in cancer biology [35, 36]. Results from RNA-Seq also show that ACSL4 overexpression caused a strong reduction in the expression of WIF1 and an increase in the expression of WNT6 and WNT10A. In agreement with our results, a requirement for ACSL4 and the involvement of GSK3 has recently been demonstrated in dorsoventral pattering of zebrafish embryo [37] and embryogenesis and neurogenesis in *Drosophila* [38, 39]. These results show that ACSL4 works through the inhibition of AKT-dependent GSK3 activity by increasing its phosphorylation. And, given the interplay between morphogenic signals in developing embryos, the interaction of these pathways has been suggested to be involved in cancer.

An important concern in the field is whether cancer can survive by acquiring adaptations that allow mTORC1 to continue signaling in a poor nutrient and oxygen environment. Because deprivation of energy, oxygen and nutrients is common in the microenvironment of tumors, cancer cells which are insensitive to these types of stress may have a selective growth advantage. Consistent with this idea, *tsc2*-deficient cells undergo apoptosis in glucose-free medium, a response suppressed by rapamycin [12].

mTOR also drives protein synthesis by regulating ribosome biogenesis. In yeast, mTORC1 activity promotes the synthesis of ribosomal proteins, which in higher organisms may additionally or alternatively involve regulating ribosome assembly [12]. As shown in Supplementary Tables S1 and S2 ACSL4 expression regulates genes associated with ribosome biogenesis and ribosome assembly. These results suggest that the expression of ACSL4 regulates mTOR activity and cell growth from diverse signals and may also control mTOR activity by regulating its upstream signaling and effectors. In this context, p70S6K is one of the major downstream components of the mTOR signaling pathway. Phospho-p70S6K (Thr389) was one of the phosphoproteins present in high levels in the MCF-7 Tet-Off/ACSL4 and T47D ACSL4 cells and in low levels in MDA MB 231 shACSL4 cells, which indicates that p70S6K is regulated by ACSL4. Phospho-S6 protein (Ser235/236) was also present at similar levels in these cell lines, once again showing a direct correlation between the regulation of mTOR activity by ACSL4 and the marked increase in mTORC1 effectors.

In many cell types, activation of mTORC1 signaling strongly represses PI3K-AKT signaling upstream in the PI3K. For example, loss of TSC1/2 function results in decreased AKT phosphorylation. One mechanism by which this occurs appears to be through p70S6K-dependent downregulation of IRS1. When mTORC1 is active, phospho-p70S6K directly phosphorylates and inhibits IRS1. This may be the case in the action of ACSL4, as there is a regulation of IRS1. In addition, ACSL4 increases the phosphorylation of TSC1/2 complex and thus inactivates it. TSC1/2 could therefore be inactivated by several mechanisms (including AKT, extracellular-signal-regulated kinase 1/2 (ERK1/2) and p70S6K — and thus, activate mTORC1.

Studies defining mTORC2 cellular functions and signaling have lagged behind, although the finding that mTORC2 directly phosphorylates AKT adds a new twist in the consideration of the role of mTOR in cancer. mTORC2 also includes the mLST8 protein but, in contrast with mTORC1, contains Rictor rather than Raptor. ACSL4 expression increases the phosphorylation of Rictor and also of AKT in Ser473. Phosphorylation of Ser473 in C-terminal hydrophobic motif is necessary for full activation of AKT. Several kinases have been proposed to fulfill the role of the AKT (Ser473) kinase/PDK2 kinase. mTOR was added to the list as a result of loss-of-function RNAi experiments coupled with *in vitro* biochemistry in *Drosophila* and in human cancer cells [18]. This study and ensuing work in human adipocytes and *Dictostylium* show that depletion of Rictor or mTOR, but not Raptor, dramatically reduce AKT (Ser473) phosphorylation [18, 40]. In *Dictostylium* PIA, the ortholog of Rictor physically interacts in a complex and, when mutated, induces similar phenotype or impaired AKT activation. Subsequent biochemical studies in mammalian and *Drosophila* culture cells confirm these observations [41]. These findings provide strong genetic evidence in mammals to substantiate the claim that mTORC2 directly regulates AKT (Ser473). Expression of ACSL4 increases AKT (Ser473) levels and the phosphorylation of Rictor. These results strongly support the concept that ACSL4 acts on the two components of mTOR, the rapamycin and nutrient-sensitive multiprotein complex and also the nutrient-insensitive mTOR-containing complex, and may help explain the strong capacity of the sole transfection of ACSL4 to change the phenotype of breast cancer cells as previously described [4]. Taken together, these findings show that ACSL4 is a new regulator of mTOR activity and up and downstream molecules of mTOR. Therefore, together with phospho-p70S6K, phospho-S6 and phospho-4E-BP1, ACSL4 appears to be a useful predictor of mTOR activity and whether breast tumors will respond to the inhibition of mTOR.

Experiments were also performed to go deeper into the mechanisms by which ACSL4 may regulate mTOR activity. ACSL4 is an important enzyme that plays a crucial role in the metabolism of AA. Particularly, we have demonstrated that ACSL4 promotes cell growth and tumor progression *in vivo* through the release of AA and its metabolization to lipoxygenase products [4]. Our results show that the lipoxygenase pathway is in part involved in the activation of mTOR. AA levels have been recently shown to strongly correlate with the signaling activity of mTORC1 and mTORC2 in human breast tumor tissues. In human breast cancer cells, AA effectively activates both mTOR complexes and its effect is mediated by lipoxygenase but not cicloxygenase metabolites [42]. In addition, it has been reported that AA induces FAK activation and migration in MDA-MB-231 cells [43]. Our RPPA results also show that ACSL4 increases FAK phosphorylation on Tyr397 and, therefore, FAK activity. We have also previously described that ACSL4 is a key enzyme in the regulation of lipoxygenase and lipoxygenase metabolites of AA in physiological conditions such as the regulation of steroidogenesis and in pathological conditions such as cancer. However, further work is needed to better understand the mechanism underlying the regulation of the different signals triggered by ACSL4 expression to regulate mTOR activity.

Despite knowing about mTOR for nearly 15 years, we are just beginning to appreciate the complexity of the mTOR network and to be able to use this pathway as an efficient therapeutic target. Although clinical results have been obtained with three prototypes of mTOR inhibitors, all rapamycin-analogs, clinical updates unfortunately indicate that rapamycin shows promising results against only few types of cancer. Overall, the therapeutic response to rapamycin is highly variable.

As ACSL4 might be a novel regulator of mTOR, the combined inhibition of an upstream mechanism such as ACSL4 activity and mTOR seems to be a potential target to be used in order to avoid compensatory feedback. We show here that rapamycin and rosiglitazone, an ACSL4 inhibitor, can act in combination to inhibit cell growth in breast cancer cells. Although ACSL4 was still not related to the mTOR pathway, this drug combination has been used previously in human non-small lung carcinoma, where the inhibitory effect of rosiglitazone on cell growth was enhanced by mTOR inhibitor rapamycin [44]. Moreover, it has been shown that rosiglitazone decreases the phosphorylation of p70S6K in human non-small lung carcinoma, an effect that was not reversed by GW9662, a peroxisome proliferator-activated receptor γ (PPARγ) antagonist [44]. We show here (Figure 8B) that rosiglitazone decreased the phosphorylation of S6, GSK3αβ, AKT (Ser473) and p70S6K, an effect that was neither reversed by GW9662. Rosiglitazone has been described as a PPARγ agonist. Therefore, it could be speculated that the effect of rosiglitazone in these experiments could be due to its activation of PPARγ and not to ACSL4 inhibition. However, the results described above, along with our findings showing that the effects of rosiglitazone on breast cancer growth *in vitro* and *in vivo* are similar to those obtained with the specific inhibition of ACSL4 by doxycycline treatment in MCF-7 Tet-Off/ACSL4 cells [4], strongly suggest that rosiglitazone effects are due to the inhibition of ACSL4 activity.

It has been suggested that the increased activation of mTOR, possibly through the PI3K/AKT pathways, plays a role in the endocrine resistance exhibited by some ERα+ breast cancer cells, and that the inhibition of mTOR signaling with rapamycin could restore sensitivity to 4-OHTAM, an inhibitor of the estrogen pathway, in laboratory models of resistance [45]. In agreement with these results, we also show that rosiglitazone can be used in combination with tamoxifen to inhibit cell growth in MCF-7 Tet-Off/ACSL4 cells. As an additional significant finding, a combination of rosiglitazone and 4-OHTAM was also effective in inhibiting cell proliferation and tumor growth in cells that do not express ER and overexpress ACSL4, such as the very aggressive triple negative breast cancer cell line MDA-MB-231.

Therefore, as ACSL4 regulates ERα expression and the mTOR pathway, our results provide evidence that ACSL4 could be also an interesting target, in combination with 4-OHTAM, to restore tumor hormone dependence in tumors with poor prognosis and low survival rates. Moreover, as the expression of ACSL4 is negatively regulated by estrogen and in turn regulates the levels of ERα, the presence of ACSL4 could be a prognostic factor for hormone resistance in ERα-positive breast cancer tissues that begin to express it. A combined therapy of ACSL4 and ERα inhibitors could thus be very useful in actually preventing the appearance of hormone resistance.

The possibility of using an ASCL4 inhibitor together with rapamycin may provide a useful combinatory therapy to target new types of cancer, including breast cancer. And, accordingly, designing effective combination therapies using ACSL4 and mTOR inhibitors together with agents targeting key molecular elements involved in breast cancer relies heavily on the identification of predictive markers that may provide the basis for patient therapy. Collectively, these findings may change the view of the pathological role mTOR plays in cancer and open doors to new therapeutic strategies.

## MATERIALS AND METHODS

### Materials

Dulbecco's modified Eagle medium (DMEM), penicillin-streptomycin solution and trypsin-EDTA were purchased from GIBCO, Invitrogen Corporation (Grand Island, NY, USA). Fetal calf serum was from PAA laboratories GmbH (Pasching, Austria). Doxycycline, 3-(4,5-dimethyl-2-thiazolyl)-2,5-diphenyl-2H-tetrazoliumbromide) (MTT) and 4-hydroxytamoxifen (4-OHTAM) were purchased from Sigma Chemical Co. (St. Louis, MO, USA). Rapamycin was obtained from Cayman Chemical Company (Michigan, IL, USA). GW9662 was obtained from Tocris Bioscience (Bristol, UK). Monoclonal mouse anti-GSK-3α/β and polyclonal rabbit anti-ERα antibodies were from Santa Cruz Biotechnology, Inc. (Dallas, TX, USA). Polyclonal rabbit phospho-GSK-3α/β (Ser21/9), phospho-p70 S6 kinase (Thr398) and phospho-S6 ribosomal protein (Ser235/236) antibodies were from Cell Signaling Technology (Boston, MA, USA). Phospho-AKT (Ser473) and phospho-Rictor (Thr1135) rabbit monoclonal antibodies were purchased from Cell Signaling Technology (Boston, MA, USA). Horseradish peroxidase-conjugated goat anti-rabbit and goat-anti-mouse secondary antibodies and Immun-Blot polyvinylidene fluoride membrane was from Bio-Rad Laboratories (Hercules, CA, USA). Enhanced chemiluminescence (ECL) was from GE Healthcare (Buckinghamshire, UK). Direct-zol RNA kit was from Zymo Research (Irvine, CA, USA). Sterile and plastic material for tissue culture was from Orange Scientific (Braine-l'Alleud, Belgium). 5-bromo-2′-deoxyuridine (BrdU) cell proliferation ELISA kit was from Roche Diagnostics, Basel, Switzerland). All other reagents were of the highest grade available.

### Cell culture

Human breast cancer cell lines MDA-MB-231, MCF-7 and T47D were generously provided by Dr. Vasilios Papadopoulos (Research Institute of the McGill University Health Centre, Montreal, Canada) and obtained from the Lombardi Comprehensive Cancer Center (Georgetown University Medical Center, Washington D.C., USA). The tetracycline-repressible MCF-7 cell lines, designated MCF-7 Tet-Off empty vector, and MCF-7 Tet-Off-induced repression of ACSL4, designated MCF-7 Tet-Off/ACSL4 were obtained previously in the laboratory [6].

For transfection of MDA-MB-231 and T47D, cells were seeded the day before and grown up to 80% confluence. Transfection was performed in Opti-MEM medium with Lipofectamine 2000 reagent (Invitrogen) using the pSUPER.retro plasmid (OligoEngine, Seattle, WA, USA) containing ACSL4 shRNA (AAGATTATTCTGTGGATGA) for the MDA-MB-231 cells and the pcDNA3.1(+) (Invitrogen) plasmid containing ACSL4 cDNA for the T47D cells. The respective empty vectors were used as controls. Transfection efficiency was estimated by counting fluorescent cells transfected with the pRc/CMVi [11] plasmid containing the enhanced form of green fluorescent protein. Forty-eight hours after transfection, cells were selected in media containing 500 μg/ml G418 and 1 μg/ml Puromycin for 1 month and then collected for biochemical and cellular assays. T47D and MDA-MB-231 transfected cell lines were designated T47D-ACSL4, T47D empty vector, MDA-MB-231 shRNA-ACSL4, and MDA MB 231 mock, respectively.

### Reverse phase protein assay (RPPA)

RPPA was performed in the RPPA Core Facility - Functional Proteomics from MD –Anderson Cancer Center, University of Texas, USA. Cellular proteins were denatured by 1% SDS (with β-mercaptoethanol) and diluted in five 2-fold serial dilutions in dilution buffer (lysis buffer containing 1% SDS). Serial diluted lysates were arrayed on nitrocellulose-coated slides (Grace Biolab) by Aushon 2470 Arrayer (Aushon BioSystems). Total 5808 array spots were arranged on each slide including the spots corresponding to positive and negative controls prepared from mixed cell lysates or dilution buffer, respectively. Each slide was probed with a validated primary antibody plus a biotin-conjugated secondary antibody. Only antibodies with a Pearson correlation coefficient between RPPA and western blotting greater than 0.7 were used in RPPA studies. Antibodies with a single or dominant band on western blotting were further assessed by direct comparison to RPPA using cell lines with differential protein expression or modulated with ligands/inhibitors or siRNA for phospho- or structural proteins, respectively. The signal obtained was amplified using a Dako Cytomation–catalyzed system (Dako) and visualized by DAB colorimetric reaction. The slides were scanned, analyzed, and quantified using a customized-software Microvigene (VigeneTech Inc.) to generate spot intensity. Each dilution curve was fitted with a logistic model (“Supercurve Fitting” developed by the Department of Bioinformatics and Computational Biology in MD Anderson Cancer Center) [46]. This fits a single curve using all the samples (i.e., dilution series) on a slide with the signal intensity as the response variable and the dilution steps as independent variables. The fitted curve is plotted with the signal intensities – both observed and fitted - on the y-axis and the log2-concentration of proteins on the x-axis for diagnostic purposes. The protein concentrations of each set of slides were then normalized by median polish, which was corrected across samples by the linear expression values using the median expression levels of all antibody experiments to calculate a loading correction factor for each sample. The list of antibodies used in the RPPA analysis is shown in Supplementary Table S3.

### RNA-Seq sample preparation and sequencing

For each cell line, total RNA was extracted by Direct-zol RNA kit (Zymo Research, Irvine, CA, USA). RNA quality was assessed by agarose gel electrophoresis (visual absence of significant 28S and 18S rRNA degradation) and by spectrophotometry. RNA-Seq was performed by Zymo Research facility performing PolyA enrichment of the RNA samples. HiSeq 2 × 50 bp paired-end reads from RNA-Seq of a human normal-tumor pair samples were analyzed first using the TopHat and Cufflinks software. TopHat (v2.0.6) was utilized for alignment of short reads to GRCh37, Cufflinks (v2.0.2) for isoform assembly and quantification, and commeRbund (v2.0.0) for visualization of differential analysis. Default parameters were used. The RNA-Seq quality control was performed using Dispersion, Volcano, MA, Density, PCA, Scatter and Box plots.

### DAVID and IPA analysis

To identify the statistically significant biological functions and signaling pathways affected by the genes differentially expressed in our comparisons, we used Database for Annotation, Visualization and Integrated Discovery (DAVID) [11] and Ingenuity Pathways Analysis (IPA; Ingenuity Systems, Inc) [25]. IPA is the largest curated database and analysis system for understanding the signaling and metabolic pathways, molecular networks, and biological processes that are most significantly changed in a dataset of interest.

Ranking and significance of the biofunctions and the canonical pathways were tested by the *p*-value. Additionally, canonical pathways were ordered by the ratio (number of genes from the input data set that map to the pathway divided by the total number of molecules that exist in the canonical pathway).

### Cell proliferation assay

Cell proliferation was measured by BrdU incorporation and MTT assays, as previously described [6, 47].

### Western blot

Western blot was performed as previously described [6]. Appropriate dilutions of primary antibodies were used as recommended by the manufacturer.

### Nude mouse xenograft model

The experimental design followed a well-established female nude mouse model [53]. MDA-MB-231 cells (5 × 10^6^ cells) mixed with Matrigel Matrix (BD Biosciences) were injected into the right flank of female Foxn1 nu/nu Balb/c athymic nude mice, aged 6–8 weeks, and allowed to form tumors. Tumors were measured with callipers every other day (length and width) and the mice weighed. Mice were provided with free access to food, water and bedding at all time and were housed with a 12 h light/dark cycle in filter top cages containing a maximum of six mice per cage. Tumor volumes (mm^3^) were calculated by the formula: π/6 × width^2^(mm^2^) × length (mm) as previously described [53]. The experiment was terminated as previously described [54] in accordance with institutionally approved guidelines.

### Ethics statement

This study was carried out in strict accordance with the recommendations in the Guide for the Care and Use of Laboratory Animals of the National Institutes of Health. The protocol was approved by the Institutional Ethical Commitee from the School of Medicine, University of Buenos Aires (ID:093/10 CD, Shool of Medicine).

### *In vivo* therapy of solid tumors in mice

For MDA-MB-231 tumor xenografts, four days after cell injection the tumor-bearing mice were randomized into the following four groups (five animals per group) and received inhibitor treatment for 25 consecutive days.

The dose of the ACSL4 inhibitor used was calculated taking into account the minimal dose required for each individual inhibitor to produce a significant effect in the xenograft MDA-MB-231 model as previously described [4]. Unitary doses were: rosiglitazone (0.6 mg/day) and tamoxifen (0.50 mg/day). In all cases, drugs were administered once a day by ip injection for rosiglitazone and tamoxifen was given through oral gavage during 25 days.

Group 1 (MDA-MB-231 cell xenografts treated with vehicle), Group 2 (MDA-MB-231 cell xenografts treated with tamoxifen), Group 3 (MDA-MB- 231 cell xenografts treated with rosiglitazone), Group 4 (MDA-MB-231 cell xenografts treated with a combination of the two drugs at the same doses used for single-drug treatments).

Animals were maintained in pathogen-free conditions and procedures were performed in accordance with recommendations for the proper use and care of laboratory animals. Tumors were measured as described above. Individual animal weights were recorded before and after treatment.

### Statistical analysis

Data analysis was performed using GraphPad Prism Software 5.01 (La Jolla, CA, USA). Statistical significance was determined by analysis of variance (ANOVA) followed by Tukey-Kramer Multiple Comparison Test. Tumor response to treatment was compared using two-way ANOVA.

## SUPPLEMENTARY TABLES


