# Supplementary material for: Acyl-CoA synthetase-4, a new regulator of mTOR and a potential therapeutic target for enhanced estrogen receptor function in receptor-positive and -negative breast cancer
Source: Oncotarget. 2015 Oct 19;6(40):42632–50. doi: 10.18632/oncotarget.5822 (PMC4767459; doi:10.18632/oncotarget.5822)
Supplement: Supplementary file 1 [file oncotarget-06-42632-s001.pdf]

SUPPLEMENTARY TABLES

Supplementary Table S1: ACSL4-regulated genes related to the mTOR pathway

Gene Symbol	Gene Name
RPS25	ribosomal protein S25
RPTOR	regulatory associated protein of MTOR, complex 1
RPS3A	ribosomal protein S3A
RPS6	ribosomal protein S6
RPS16	ribosomal protein S16
RPS28	ribosomal protein S28
MTOR	mechanistic target of rapamycin (serine/threonine kinase)
RPS9	ribosomal protein S9
AKT1	v-akt murine thymoma viral oncogene homolog 1
EIF4G3	eukaryotic translation initiation factor 4 gamma, 3
EIF4EBP1	eukaryotic translation initiation factor 4E binding protein 1
NAPEPLD	N-acyl phosphatidylethanolamine phospholipase D
RHOV	ras homolog family member V
EIF3H	eukaryotic translation initiation factor 3, subunit H
PLD1	phospholipase D1, phosphatidylcholine-specific
RPS2	ribosomal protein S2
EIF3I	eukaryotic translation initiation factor 3, subunit I
EIF4A2	eukaryotic translation initiation factor 4A2
RPS20	ribosomal protein S20
MAPK1	mitogen-activated protein kinase 1
PPP2R5D	protein phosphatase 2, regulatory subunit B', delta
RHOB	ras homolog family member B
FKBP1A	FK506 binding protein 1A, 12kDa
RHOA	ras homolog family member A
ULK1	unc-51-like kinase 1 (C. elegans)
EIF3G	eukaryotic translation initiation factor 3, subunit G
RPS18	ribosomal protein S18
EIF3F	eukaryotic translation initiation factor 3, subunit F
RPS26	ribosomal protein S26
RPS10	ribosomal protein S10
TSC1	tuberous sclerosis 1
PRKCD	protein kinase C, delta
HRAS	v-Ha-ras Harvey rat sarcoma viral oncogene homolog
RPS23	ribosomal protein S23

(*Continued*)

Gene Symbol	Gene Name
RPS19	ribosomal protein S19
RPS12	ribosomal protein S12
RHEB	Ras homolog enriched in brain
RHOU	ras homolog family member U
RPSA	ribosomal protein SA
ATG13	autophagy related 13
PRKAA1	protein kinase, AMP-activated, alpha 1 catalytic subunit
PRKAB1	protein kinase, AMP-activated, beta 1 non-catalytic subunit
STK11	serine/threonine kinase 11
INSR	insulin receptor
PPP2R4	protein phosphatase 2A activator, regulatory subunit 4
VEGFA	vascular endothelial growth factor A
PIK3C3	phosphatidylinositol 3-kinase, catalytic subunit type 3
EIF3A	eukaryotic translation initiation factor 3, subunit A
EIF3L	eukaryotic translation initiation factor 3, subunit L
AKT2	v-akt murine thymoma viral oncogene homolog 2
EIF3J	eukaryotic translation initiation factor 3, subunit J
PPP2R2A	protein phosphatase 2, regulatory subunit B, alpha
RHOQ	ras homolog family member Q
PPP2R5A	protein phosphatase 2, regulatory subunit B', alpha
PRKD1	protein kinase D1
RPS4X	ribosomal protein S4, X-linked
DGKZ	diacylglycerol kinase, zeta
RHOG	ras homolog family member G
RPS8	ribosomal protein S8
EIF3M	eukaryotic translation initiation factor 3, subunit M
IRS1	insulin receptor substrate 1
PRKCZ	protein kinase C, zeta
PRKAG2	protein kinase, AMP-activated, gamma 2 non-catalytic subunit
RPS3	ribosomal protein S3
RPS27A	ribosomal protein S27a
EIF4A1	eukaryotic translation initiation factor 4A1
AKT1S1	AKT1 substrate 1 (proline-rich)
EIF4A3	eukaryotic translation initiation factor 4A3
MAPKAP1	mitogen-activated protein kinase associated protein 1
PLD2	phospholipase D2
PIK3R2	phosphoinositide-3-kinase, regulatory subunit 2 (beta)

(*Continued*)

Gene Symbol	Gene Name
RPS14	ribosomal protein S14
RPS21	ribosomal protein S21
RPS6KA1	ribosomal protein S6 kinase, 90kDa, polypeptide 1
RPS5	ribosomal protein S5
PIK3CA	phosphatidylinositol-4,5-bisphosphate 3-kinase, catalytic subunit alpha
RHOF	ras homolog family member F (in filopodia)
EIF3D	eukaryotic translation initiation factor 3, subunit D
PLD6	phospholipase D family, member 6
PIK3C2B	phosphatidylinositol-4-phosphate 3-kinase, catalytic subunit type 2 beta
PIK3R3	phosphoinositide-3-kinase, regulatory subunit 3 (gamma)
RPS6KB1	ribosomal protein S6 kinase, 70kDa, polypeptide 1
RPS7	ribosomal protein S7
PRKD3	protein kinase D3
RRAS	related RAS viral (r-ras) oncogene homolog
TSC2	tuberous sclerosis 2
PRR5L	proline rich 5 like
DDIT4	DNA-damage-inducible transcript 4
EIF3E	eukaryotic translation initiation factor 3, subunit E
EIF4B	eukaryotic translation initiation factor 4B
RPS27	ribosomal protein S27
RPS24	ribosomal protein S24
RHOT1	ras homolog family member T1
RPS15	ribosomal protein S15

Supplementary Table S2: ACSL4-regulated genes related to the eIF4 and p70S6K signaling

Gene Symbol	Gene Name
RPS11	ribosomal protein S11
RPS25	ribosomal protein S25
EIF1	eukaryotic translation initiation factor 1
RPS3A	ribosomal protein S3A
ITGA3	integrin, alpha 3 (antigen CD49C, alpha 3 subunit of VLA-3 receptor)
RPS6	ribosomal protein S6
RPS16	ribosomal protein S16
RPS28	ribosomal protein S28
MTOR	mechanistic target of rapamycin (serine/threonine kinase)
RPS9	ribosomal protein S9
EIF2S1	eukaryotic translation initiation factor 2, subunit 1 alpha, 35kDa
ITGA5	integrin, alpha 5 (fibronectin receptor, alpha polypeptide)
AKT1	v-akt murine thymoma viral oncogene homolog 1
EIF4EBP1	eukaryotic translation initiation factor 4E binding protein 1
EIF4G3	eukaryotic translation initiation factor 4 gamma, 3
EIF4EBP2	eukaryotic translation initiation factor 4E binding protein 2
EIF3H	eukaryotic translation initiation factor 3, subunit H
RPS2	ribosomal protein S2
EIF3I	eukaryotic translation initiation factor 3, subunit I
EIF4A2	eukaryotic translation initiation factor 4A2
RPS20	ribosomal protein S20
MAPK1	mitogen-activated protein kinase 1
PABPC1	poly(A) binding protein, cytoplasmic 1
PPP2R5D	protein phosphatase 2, regulatory subunit B’, delta
EIF1AX	eukaryotic translation initiation factor 1A, X-linked
EIF3G	eukaryotic translation initiation factor 3, subunit G
RPS18	ribosomal protein S18
MAPK14	mitogen-activated protein kinase 14
EIF3F	eukaryotic translation initiation factor 3, subunit F
SOS1	son of sevenless homolog 1 (Drosophila)
RPS26	ribosomal protein S26
RPS10	ribosomal protein S10
PAIP2	poly(A) binding protein interacting protein 2
HRAS	v-Ha-ras Harvey rat sarcoma viral oncogene homolog
RPS23	ribosomal protein S23
RPS19	ribosomal protein S19

(*Continued*)

Gene Symbol	Gene Name
RPS12	ribosomal protein S12
EIF2B2	eukaryotic translation initiation factor 2B, subunit 2 beta, 39kDa
EIF2S3	eukaryotic translation initiation factor 2, subunit 3 gamma, 52kDa
RPSA	ribosomal protein SA
PAIP1	poly(A) binding protein interacting protein 1
PPP2R4	protein phosphatase 2A activator, regulatory subunit 4
PIK3C3	phosphatidylinositol 3-kinase, catalytic subunit type 3
EIF3A	eukaryotic translation initiation factor 3, subunit A
EIF3L	eukaryotic translation initiation factor 3, subunit L
ITGA2	integrin, alpha 2 (CD49B, alpha 2 subunit of VLA-2 receptor)
AKT2	v-akt murine thymoma viral oncogene homolog 2
EIF3J	eukaryotic translation initiation factor 3, subunit J
PPP2R2A	protein phosphatase 2, regulatory subunit B, alpha
PPP2R5A	protein phosphatase 2, regulatory subunit B', alpha
RPS4X	ribosomal protein S4, X-linked
RPS8	ribosomal protein S8
EIF3M	eukaryotic translation initiation factor 3, subunit M
IRS1	insulin receptor substrate 1
PRKCZ	protein kinase C, zeta
EIF2B4	eukaryotic translation initiation factor 2B, subunit 4 delta, 67kDa
RPS27A	ribosomal protein S27a
RPS3	ribosomal protein S3
EIF4A1	eukaryotic translation initiation factor 4A1
EIF4A3	eukaryotic translation initiation factor 4A3
RPS14	ribosomal protein S14
PIK3R2	phosphoinositide-3-kinase, regulatory subunit 2 (beta)
RPS21	ribosomal protein S21
RPS5	ribosomal protein S5
PIK3CA	phosphatidylinositol-4,5-bisphosphate 3-kinase, catalytic subunit alpha
EIF3D	eukaryotic translation initiation factor 3, subunit D
PIK3C2B	phosphatidylinositol-4-phosphate 3-kinase, catalytic subunit type 2 beta
EIF2B1	eukaryotic translation initiation factor 2B, subunit 1 alpha, 26kDa
PIK3R3	phosphoinositide-3-kinase, regulatory subunit 3 (gamma)
RPS7	ribosomal protein S7
RPS6KB1	ribosomal protein S6 kinase, 70kDa, polypeptide 1
RRAS	related RAS viral (r-ras) oncogene homolog
EIF3E	eukaryotic translation initiation factor 3, subunit E

(*Continued*)

Gene Symbol	Gene Name
MAPK11	mitogen-activated protein kinase 11
RPS27	ribosomal protein S27
RPS24	ribosomal protein S24
RPS15	ribosomal protein S15
GRB2	growth factor receptor-bound protein 2

Supplementary Table S3: List of antibodies used in RPPA

Official Antibody Name	Gene Name
14–3-3-beta-R-V	YWHAB
14–3-3-epsilon-M-C	YWHAE
14–3-3-zeta-R-V	YWHAZ
4E-BP1-R-V	EIF4EBP1
4E-BP1_pS65-R-V	EIF4EBP1
4E-BP1_pT37_T46-R-V	EIF4EBP1
53BP1-R-V	TP53BP1
A-Raf-R-V	ARAF
ACC_pS79-R-V	ACACA ACACB
ACC1-R-E	ACACA
ACVRL1-R-C	ACVRL1
ADAR1-M-V	ADAR
Akt-R-V	AKT1 AKT2 AKT3
Akt_pS473-R-V	AKT1 AKT2 AKT3
Akt_pT308-R-V	AKT1 AKT2 AKT3
AMPK-alpha-R-C	PRKAA1
AMPK-alpha_pT172-R-V	PRKAA1
Annexin-I-M-V	ANXA1
Annexin-VII-M-V	ANXA7
AR-R-V	AR
ARHI-M-C	DIRAS3
ATM-R-V	ATM
ATM_pS1981-R-V	ATM
ATP5H-M-C	ATP5H
ATR-R-C	ATR
b-Catenin-R-V	CTNNB1
b-Catenin_pT41_S45-R-V	CTNNB1
B-Raf-M-C	BRAF
B-Raf_pS445-R-V	BRAF
Bad_pS112-R-V	BAD
Bak-R-C	BAK1
BAP1-M-V	BAP1
Bax-R-V	BAX
Bcl-xL-R-V	BCL2L1
Bcl2-M-V	BCL2
Beclin-G-C	BECN1

(*Continued*)

Official Antibody Name	Gene Name
Bid-R-C	BID
Bim-R-V	BCL2L11
BRCA2-R-C	BRCA2
c-Jun_pS73-R-V	JUN
c-Kit-R-V	KIT
c-Met-M-QC	MET
c-Met_pY1234_Y1235-R-V	MET
c-Myc-R-C	MYC
C-Raf-R-V	RAF1
C-Raf_pS338-R-V	RAF1
Caspase-7-cleaved-R-C	CASP7
Caspase-8-M-QC	CASP8
Caveolin-1-R-V	CAV1
CD29-M-V	ITGB1
CD31-M-V	PECAM1
CD49b-M-V	ITGA2
CDK1-R-V	CDC2-CDK1
Chk1-M-C	CHEK1
Chk1_pS345-R-C	CHEK1
Chk2-M-V	CHEK2
Chk2_pT68-R-C	CHEK2
Claudin-7-R-V	CLDN7
Collagen-VI-R-V	COL6A1
Complex-II-Subunit-M-V	SDHA
Cox-IV-M-C	COX4I1
Cox2-R-C	PTGS2
Cyclin-B1-R-V	CCNB1
Cyclin-D1-R-V	CCND1
Cyclin-E1-M-V	CCNE1
Cyclophilin-F-M-V	PPIF
DJ1-R-V	PARK7
Dvl3-R-V	DVL3
E-Cadherin-R-V	CDH1
E2F1-M-V	E2F1
eEF2-R-C	EEF2
eEF2K-R-V	EEF2K
EGFR-R-V	EGFR

(*Continued*)

Official Antibody Name	Gene Name
EGFR_pY1068-R-C	EGFR
EGFR_pY1173-R-V	EGFR
eIF4E-R-V	EIF4E
eIF4G-R-C	EIF4G1
ER-alpha-R-V	ESR1
ER-alpha_pS118-R-V	ESR1
ERCC1-M-V	ERCC1
Ets-1-R-V	ETS1
FAK-R-E	PTK2
FAK_pY397-R-V	PTK2
FASN-R-V	FASN
Fibronectin-R-V	FN1
FoxM1-R-V	FOXM1
FoxO3a-R-C	FOXO3
FoxO3a_pS318_S321-R-C	FOXO3
G6PD-M-V	G6PD
Gab2-R-V	GAB2
GAPDH-M-C	GAPDH
GATA3-M-V	GATA3
GCN5L2-R-V	KAT2A
GPBB-R-V	PYGB
GSK-3ab-M-V	GSK3A GSK3B
GSK-3ab_pS21_S9-R-V	GSK3A GSK3B
GSK-3b_pS9-R-V	GSK3B
Gys-R-V	GYS1
Gys_pS641-R-V	GYS1
HER2-M-V	ERBB2
HER2_pY1248-R-C	ERBB2
HER3-R-V	ERBB3
HER3_pY1289-R-C	ERBB3
Heregulin-R-V	NRG1
HIAP-R-C	BIRC2
Histone-H3-R-V	H3F3A H3F3B
IGF1R-beta-R-V	IGF1R
IGFBP2-R-V	IGFBP2
INPP4b-R-V	INPP4B
IRS1-R-V	IRS1

(*Continued*)

Official Antibody Name	Gene Name
JAB1-M-C	COPS5
JNK_pT183_Y185-R-V	MAPK8
JNK2-R-C	MAPK9
Lck-R-V	LCK
MAPK_pT202_Y204-R-V	MAPK1 MAPK3
Mcl-1-R-V	MCL1
MDM2_pS166-R-V	MDM2
MEK1-R-V	MAP2K1
MEK1_pS217_S221-R-V	MAP2K1 MAP2K2
MEK2-R-V	MAP2K2
Merlin-R-C	NF2
MIG6-M-V	ERRFI1
MSH2-M-V	MSH2
MSH6-R-C	MSH6
mTOR-R-V	MTOR
mTOR_pS2448-R-C	MTOR
Myosin-11-R-V	MYH11
Myosin-IIa_pS1943-R-V	MYH9
N-Cadherin-R-V	CDH2
N-Ras-M-V	NRAS
NAPSIN-A-R-C	NAPSA
NDRG1_pT346-R-V	NDRG1
NF-kB-p65_pS536-R-C	RELA
Notch1-R-V	NOTCH1
p16INK4a-R-V	CDKN2A
p21-R-V	CDKN1A
p27-Kip-1-R-V	CDKN1B
p27_pT157-R-C	CDKN1B
p27_pT198-R-V	CDKN1B
p38-alpha-M-V	MAPK14
p38-R-V	MAPK14
p38_pT180_Y182-R-V	MAPK14
p53-R-C	TP53
p70-S6K_pT389-R-V	RPS6KB1
p70-S6K1-R-V	RPS6KB1
PAI-1-M-V	SERPINE1
PARP-cleaved-M-QC	PARP1

(*Continued*)

Official Antibody Name	Gene Name
PARP1-R-V	PARP1
Paxillin-R-C	PXN
PCNA-M-C	PCNA
Pdcd-1L1-G-C	CD274
Pdcd4-R-C	PDCD4
PDGFR-beta-R-V	PDGFRB
PDK1-R-V	PDPK1
PDK1_pS241-R-V	PDPK1
PEA-15-R-V	PEA15
PEA-15_pS116-R-V	PEA15
PI3K-p110-alpha-R-C	PIK3CA
PI3K-p85-R-V	PIK3R1
PKC-alpha-M-V	PRKCA
PKC-alpha_pS657-R-C	PRKCA
PKC-beta-II_pS660-R-V	PRKCA PRKCB PRKCD PRKCE PRKCH PRKCQ
PKC-delta_pS664-R-V	PRKCD
PMS2-R-V	PMS2
Porin-M-V	VDAC1
PR-R-V	PGR
PRAS40_pT246-R-V	AKT1S1
PREX1-R-V	PREX1
PTEN-R-V	PTEN
Rab11-R-E	RAB11A RAB11B
Rab25-R-V	RAB25
Rad50-M-V	RAD50
Rad51-R-V	RAD51
Raptor-R-V	RPTOR
Rb-M-QC	RB1
Rb_pS807_S811-R-V	RB1
RBM15-R-V	RBM15
Rictor-R-C	RICTOR
Rictor_pT1135-R-V	RICTOR
RSK-R-C	RPS6KA1 RPS6KA2 RPS6KA3
S6_pS235_S236-R-V	RPS6
S6_pS240_S244-R-V	RPS6
SCD-M-V	SCD
SETD2-R-QC	SETD2

(*Continued*)

Official Antibody Name	Gene Name
SF2-M-V	SRSF1
Shc_pY317-R-V	SHC1
Smac-M-QC	DIABLO
Smad1-R-V	SMAD1
Smad3-R-V	SMAD3
Smad4-M-V	SMAD4
Snail-M-QC	SNAI1
Src-M-V	SRC
Src_pY416-R-C	SRC LYN FYN LCK YES1 HCK
Src_pY527-R-V	SRC YES1 FYN FGR
Stat3_pY705-R-V	STAT3
Stat5a-R-V	STAT5A
Stathmin-1-R-V	STMN1
Syk-M-V	SYK
TAZ-R-V	WWTR1
TFRC-R-V	TFRC
TIGAR-R-V	C12ORF5
Transglutaminase-M-V	TGM2
TSC1-R-C	TSC1
TTF1-R-V	NKX2–1
Tuberin-R-V	TSC2
Tuberin_pT1462-R-V	TSC2
TWIST-M-C	TWIST2
Tyro3-R-V	TYRO3
UBAC1-R-V	UBAC1
UGT1A-M-V	UGT1A1
UQCRC2-M-C	UQCRC2
VEGFR-2-R-V	KDR
XRCC1-R-C	XRCC1
YAP-R-E	YAP1
YAP_pS127-R-E	YAP1
YB1-R-V	YBX1
YB1_pS102-R-V	YBX1
